# Supplementary material for: Iron acquisition in the mutualistic fungus Penicillium herquei: implications of mineral elements in insect-fungus symbiosis
Source: Microbiol Spectr. 2025 Aug 7;13(9):e01051-25. doi: 10.1128/spectrum.01051-25 (PMC12403598; doi:10.1128/spectrum.01051-25)
Supplement: Supplemental figures — Fig. S1 and S2. [file spectrum.01051-25-s0001.pdf]

SUPPLEMENTAL MATERIAL

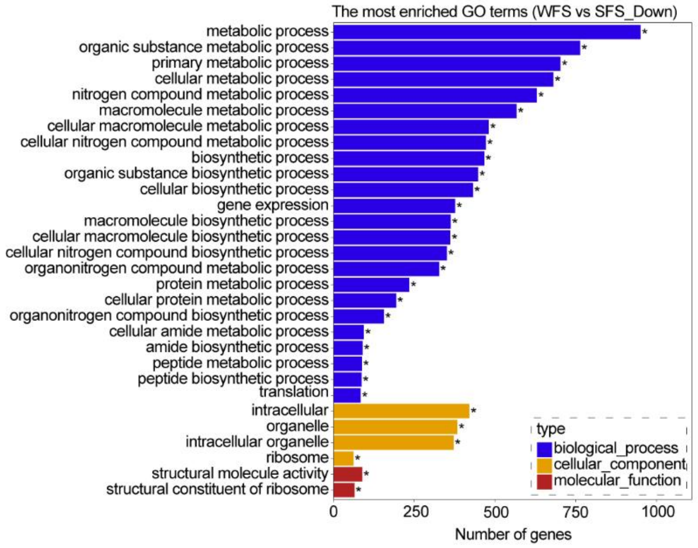

Fig. S1 GO enrichment analysis of differentially down-regulated genes in weevil-farming strain (WFS) compared to soil free-living strain (SFS) of *Penicillum herquei*.

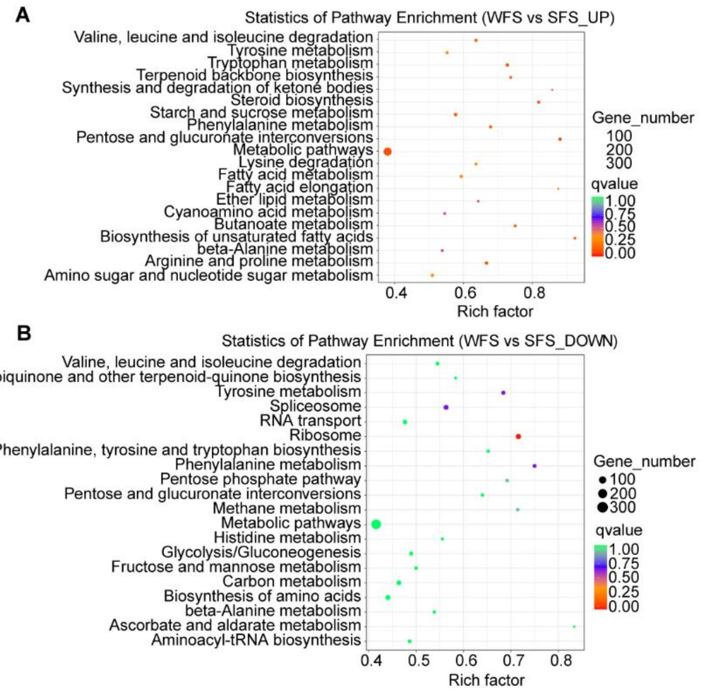

Fig. S2 Significantly enriched KEGG pathways of differentially up-regulated (**A**) and down-regulated (**B**) genes for weevil-farming strain (WFS) and soil free-living strain (SFS) of *Penicillum herquei*.
